# Measurement of Tibial Orientation Helps Select the Optimal Insert Thickness to Personalize PCL Tension in a Medial Ball-in-Socket TKA

**DOI:** 10.3390/jpm12091427

**Published:** 2022-08-31

**Authors:** Alexander J. Nedopil, Stephen M. Howell, Maury L. Hull

**Affiliations:** 1 Orthopädische Klinik König-Ludwig-Haus, Orthopädie der Universität Würzburg, 97074 Würzburg, Germany; 2 Department of Biomedical Engineering, University of California, Davis, CA 95616, USA; 3 Department of Mechanical Engineering, University of California, Davis, CA 95616, USA; 4 Department of Orthopedic Surgery, University of California Davis Medical Center, Sacramento, CA 95817, USA

**Keywords:** posterior cruciate ligament, tibial rotation, medial pivot, total knee arthroplasty, kinematic alignment

## Abstract

As the conformity of a medial ball-in-socket total knee arthroplasty (TKA) provides intrinsic anterior-posterior (A-P) stability, surgeons cannot rely on the manual examination of sagittal laxity to identify the optimal insert thickness. Instead, the present study determined whether measuring tibial axial orientation in extension and 90° flexion with an insert goniometer could identify the optimal thickness that, when implanted, provides high postoperative function. In twenty-two patients that underwent unrestricted caliper-verified kinematic alignment (KA) with a PCL retaining implant, two surgeons measured tibial orientation in extension and 90° flexion with 10, 11, 12, and 13 mm thick insert goniometers. Each TKA had one insert thickness that restored either the maximum external tibial orientation in extension, the maximum internal tibial orientation at 90° flexion, or both relative to 1 mm thinner and thicker inserts. In addition, the 6-month median [interquartile range] Forgotten Joint Score of 73 (54–87) and Oxford Knee Score of 42 (38–45) indicated high satisfaction and function. In conclusion, surgeons using a medial ball-in-socket TKA design can measure external tibial orientation in extension and internal tibial orientation at 90° flexion with an insert goniometer. Furthermore, implanting an insert with the thickness that provided the maximum orientation values resulted in high postoperative function, thereby personalizing PCL tension.

## 1. Introduction

A total knee arthroplasty (TKA) should restore the native or healthy knee’s resting length and tension of the posterior cruciate ligament (PCL) throughout the range of motion to provide stability and to not over or under constrain the knee [1]. An overtight PCL can decrease the range of motion, increase the risks of tibial component subsidence and polyethylene wear, and cause anterior tibial subluxation that can lead to pain, effusion, and impaired function [1,2,3,4,5,6].

A common assumption in TKA is that an implant design that achieves closer to native knee kinematics will provide better outcomes and lower revision rates [7]. One design gaining interest is the medial ball-in-socket insert, which provides intrinsic anterior-posterior (A-P) stability and kinematics closer to native than less-conforming designs and improves patient satisfaction and function [8,9,10,11,12,13,14]. However, due to the medial ball-in-socket insert’s intrinsic A-P stability, the surgeon cannot rely on the manual examination of sagittal laxity to select the optimal thickness of the insert.

As the medial ball-in-socket insert allows internal-external tibial rotation rather than A-P translation, a method that measures tibial axial rotation instead of sagittal laxity might be able to select the optimal insert thickness [9,10,12]. In the native knee, achieving maximum internal tibial rotation from extension to 90° flexion requires a PCL with native tension [15,16,17]. In the TKA, a slight sagittal deviation of 2°> and 2°< of the tibial component relative to the patient’s pre-arthritic slope slackened and tightened the PCL in flexion, respectively, causing a 4° loss of internal tibial rotation [18]. Hence, in TKA, the PCL should have native tension when the tibia has maximum external orientation in extension and maximal internal orientation at 90° flexion.

**Figure 1:**
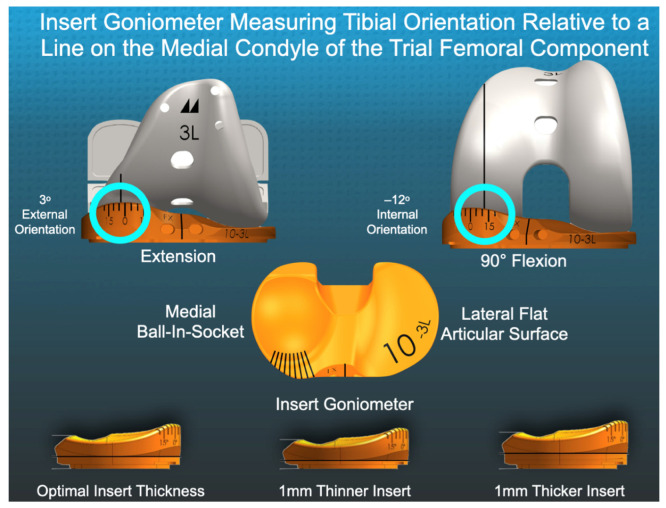
The composite shows the intersection of the sagittal reference line on the femoral trial component with the trial insert goniometer’s angular scale (blue circles), which measured 3° external orientation in extension and −12° internal orientation at 90° flexion.

The insert goniometer is an inexpensive instrument for measuring the degree of tibial orientation. The insert has an angular scale that records tibial orientation at the intersection with a sagittal line on the medial condyle of the trial femoral component [18,19] (Figure 1). The device has a repeatability of 1° and ICC values for reproducibility of 0.89 at extension and 0.87 at 90° of flexion [20].

Finally, it is necessary to provide supportive evidence that the thickness selected by the insert goniometer is ‘optimal’ by determining whether the patient reports high satisfaction and function as measured with validated follow-up patient-reported outcome scores. For example, values representing high satisfaction and function for the Forgotten Joint Score (100 best, 0 worst) of 80 and 70 points indicate negligible and minor knee function restrictions, respectively [21]. For the Oxford Knee Score (48 best, 0 worst), the excellent category range is 42–48 points [22].

Accordingly, the present study evaluated twenty-two patients who underwent unrestricted caliper-verified kinematic alignment (KA) with a medial ball-in-socket implant design and PCL retention to determine whether measurements of tibial orientation can select the optimal insert thickness. The first hypothesis was that the surgeon could identify the optimal insert thickness as the one that provided either the maximum external tibial orientation in extension, maximum internal tibial orientation at 90° flexion, or both relative to inserts 1 mm thinner and thicker. The second hypothesis was that implanting the optimal insert thickness resulted in high satisfaction and function at 6 months.

## 2. Materials and Methods

Our institutional review board approved the retrospective study (Pro00054838). Two surgeons performed unrestricted caliper-verified kinematic alignment (KA) and PCL retention with a medial ball-in-socket and flat lateral articulation using a mid-vastus exposure (GMK Sphere, Medacta International). From April 2021 to June 2021, one-time use insert goniometers were used on a first-come, first-serve basis in twenty-two patients based on the availability of sterile 3-D printed 10, 11, 12, and 13 mm thick inserts in sizes 3, 4, and 5 for left and right tibial baseplates (Figure 1). Each patient fulfilled the Centers for Medicare & Medicaid Services guidelines for medical necessity for TKA treatment including: (1) radiographic evidence of Kellgren–Lawrence Grade II to IV arthritic change or osteonecrosis; (2) any severity of clinical varus or valgus deformity; (3) and any severity of flexion contracture. Caliper measurements enabled the matching of the thickness of the femoral bone resections to the thickness of the femoral component within ±0.5 mm after correcting for wear and the saw blade’s kerf using a previously described technique [23].

The following steps using trial components determined the preliminary insert thickness. First, the surgeon placed the knee at 90° flexion and palpated the PCL to verify that it was intact. Next, the surgeon verified that the knee was hyperextended a few degrees, like the pre-arthritic knee. When the knee had a flexion contracture, either a thinner insert was used, or the posterior capsule was manually released by gently hyperextending the TKA. With the TKA in maximum extension, the surgeon verified that the varus-valgus (V-V) laxity was negligible. With the knee in 15–30° flexion, the surgeon verified a ~3–4 mm gap in the lateral compartment and a ~1 mm gap in the medial compartment. A 1 mm thinner insert was used when anterior lift-off of the insert from the trial baseplate occurred at 90° flexion, which indicated an over-tensioned PCL [4]. When necessary, the tibia was recut to accept either an 11 or 12 mm insert.

**Figure 2:**
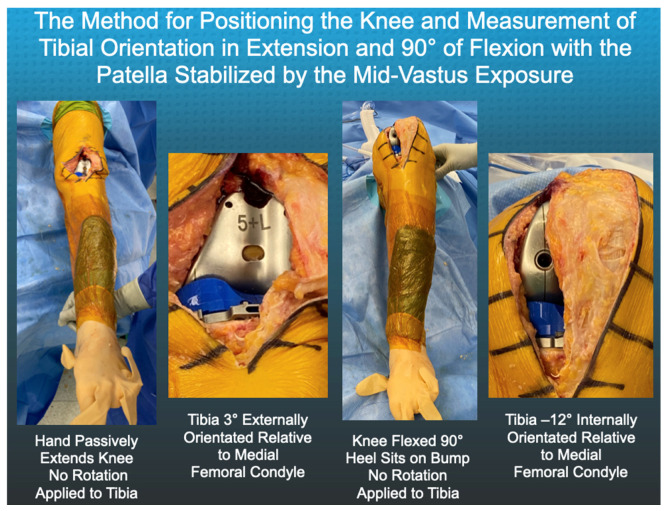
Composite shows photographs of a left leg and the steps for measuring tibial orientation with the insert goniometer. The surgeon passively extended the TKA and measured external tibial orientation of 3°. The surgeon flexed the TKA and measured −12° of internal tibial orientation at 90° flexion.

The surgeon performed the following steps using the insert goniometer to measure the tibial orientation in extension and 90° flexion (Figure 2). The surgeon reduced the patella, which was resurfaced with an anatomic component. The proximal vastus medialis, which was left attached to the quadriceps tendon by the mid-vastus exposure, promoted patella tracking in the prosthetic trochlea. The knee was passively extended by lifting the leg without applying an internal-external axial (I-E) moment to the ankle. External tibial orientation was measured with the insert goniometer (+external/−internal). The surgeon placed the knee at 90° flexion and rested the foot on the operating table and measured internal tibial orientation. These steps were performed using 10, 11, 12, and 13 mm insert goniometers. Inspection confirmed maximum extension with each insert thickness and flexion to 90°. The optimal insert thickness was the one that restored either the maximum external tibial orientation in extension, maximum internal tibial orientation at 90° flexion, or both without anterior lift-off of the insert at 90° flexion.

At 6-months follow-up, patients received a postal and electronically mailed questionnaire containing assessments of clinical outcomes scores. Each patient completed the Forgotten Joint Score (100 best, 0 worst) and Oxford Knee Score (48 best, 0 worst).

### Statistical Analysis

Data were analyzed using statistical software (JMP^®^ Pro 16.2.0, www.jmp.com, accessed on 28 March 2021, SAS, Cary, NC, USA). A single-factor repeated-measures analysis of variance with three levels (i.e., optimal insert, 1 mm thinner insert, and 1 mm thicker insert) determined whether there was a difference in mean tibial orientation in extension and at 90° flexion. For each analysis, post hoc paired *t*-tests with Bonferroni adjustment in *p*-values determined the differences between all pairs of insert thickness. Significance for the ANOVAs was *p* < 0.05 and significance for the paired *t*-tests was 0.0167 reflecting the Bonferroni adjustment. To quantify the reproducibility of the goniometric measurement, two surgeons measured the external tibial orientation in extension and tibial internal orientation at 90° flexion in 7 randomly selected patients. Software computed the intraclass correlation coefficient (ICC) for each measurement using a 2-factor analysis of variance with random effects. The first factor was the observer with two levels (surgeons 1 and 2). The second factor was the 7 patients. An ICC value of >0.9 indicates excellent agreement, and 0.75–0.90 indicates good agreement [24]. ICC values of 0.82 for the measurement of the external tibial orientation in extension and 0.87 for internal tibial orientation at 90° flexion indicated good reproducibility.

The lack of historical differences in the tibial orientation from a repeated-measures study design precluded an a priori power analysis. Instead, the study included a post hoc computation (G* Power 3.1.9.6 for Mac OS 10.7 to 12). The inputs consisted of Type I error (alpha) of 0.05, sample size of 22, number of patient groups = 1, number of external or internal orientation measurements = 3. With an error standard deviation of 3.8° and an effect size of 0.39, the corresponding power was 0.99.

## 3. Results

Descriptive statistics of the twenty-two patients’ clinical characteristics, knee range-of-motion, clinical outcome scores, Kellgren–Lawrence arthritic rating, radiographic alignment and clinical outcome scores are listed in Table 1.

In extension, the 7° mean external or screw-home tibial orientation for the optimal insert thickness was greater than the 5° and 4° for the 1 mm thinner and thicker inserts, respectively (ANOVA: *p* < 0.0001, paired *t*-test: *p* < 0.0001 for both thinner and thicker inserts). At 90° flexion, the magnitude of the −15° mean internal tibial orientation for the optimal insert thickness was greater than the magnitudes of −12° and −10° for the 1 mm thinner and thicker inserts, respectively (ANOVA: *p* < 0.0001, paired *t*-test: *p* < 0.0001 for both thinner and thicker inserts).

**Figure 3:**
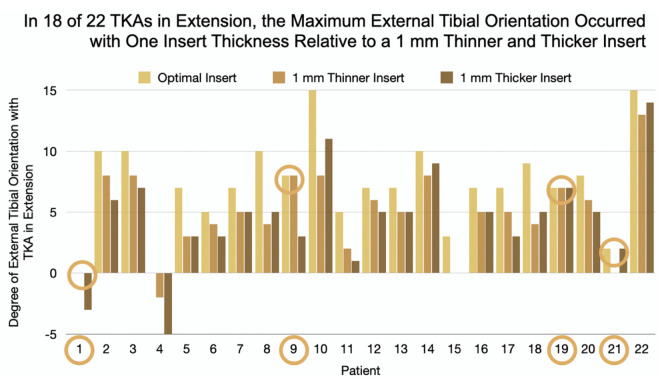
For each TKA in extension, the column graph shows the degree of external tibial orientation for the optimal and 1 mm thinner and thicker inserts. The optimal insert thickness was determined at 90° flexion in four patients because two insert thicknesses had the same maximum external orientation in extension (circled in brown).

**Figure 4:**
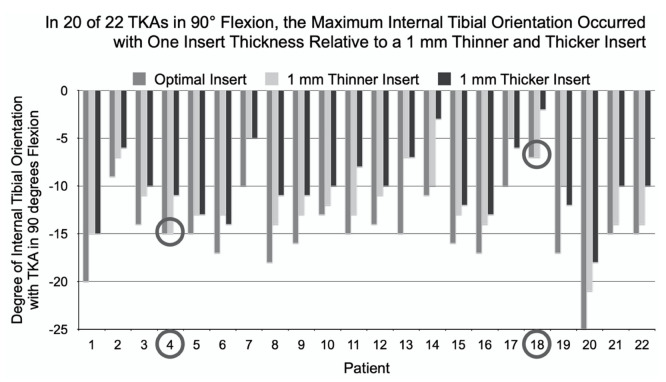
For each TKA at 90° flexion, the column graph shows the degree of internal tibial orientation for the optimal and 1 mm thinner and thicker inserts. The optimal insert thickness was determined in extension in two patients because two insert thicknesses had the same maximum internal orientation at 90° flexion (circled in gray).

In 16 of 22 (73%) patients, only one insert thickness (i.e., optimal) provided the maximum external orientation in extension and the maximum internal orientation at 90° flexion. In 4 patients (1, 9, 19, and 21), two insert thicknesses provided the same maximum external tibial orientation, so the determination of the optimal insert thickness was at 90° flexion (Figure 3). In 2 patients (4 and 18), two insert thicknesses provided the same maximum internal tibial orientation at 90° flexion; so, the determination of the optimal insert thickness was in extension (Figure 4). In 4 patients anterior lift-off occurred with the 1 mm thicker insert, which was managed by using a 1 mm thinner insert and not be recutting the tibia.

The 6-month postoperative median [interquartile range] Forgotten Joint Score was 73 (54–87) and Oxford Knee Score was 42 (38–45).

## 4. Discussion

The present study of a medial ball-in-socket insert measured axial tibial orientation as a substitute for manual A-P laxity assessment to identify the optimal insert thickness, thereby personalizing ligament and PCL tension after unrestricted caliper-verified KA TKA. One important finding was that each TKA had one (i.e., optimal) insert thickness that restored either maximum external tibial orientation in extension or maximum internal tibial orientation at 90° flexion relative to 1 mm thinner and thicker inserts. A second was that implanting the optimal insert thickness achieved high satisfaction and function at 6-months as measured by the Forgotten Joint Score and Oxford Knee Score.

The insert goniometer is inexpensive, requires little change in the surgical workflow, and allows a quantitative selection of the optimal insert thickness. When an insert increases external orientation in extension or internal orientation in 90° flexion, or both, the thickness is closer to optimal. When an insert decreases external or internal orientation or both, the thickness is farther from optimal. Selecting an insert that provides negligible V-V laxity in extension, ~3–4 mm lateral and ~1 mm medial gap in 30° flexion, and no lift-off of the insert at 90° flexion restores native tibial compartment forces by default without input from a tibial force sensor [18,19,20,23,25]. Therefore, the insert goniometer reduced the risk of selecting an insert 1 mm too thick and 1 mm too thin that could over and under tension the TKA, respectively.

Identifying the optimal insert thickness has potential kinematic benefits. In the present study, the optimal insert restored a mean of 7° of external tibial orientation in extension, which is the ‘screw home motion’ or locking mechanism of the native knee [26]. In addition, the internal tibial rotation of −17°, computed from the mean external orientation of 5° in extension and −12° of internal tibial orientation at 90° flexion, was comparable to the −14° internal tibial rotation reported in a study of five native knees during a deep knee bend [27,28]. The benefit of native screw home movement is that it tensions the lateral and medial retinacular ligaments, which assists in capturing the patella during early knee flexion before confinement in the trochlear groove [21]. Furthermore, restoring native internal tibial orientation at 90° flexion reduces the Q-angle that guides patellofemoral tracking [14,15,16]. Hence, selecting the optimal insert thickness could promote more natural patellofemoral kinematics in TKA.

According to published criteria, the insert goniometer’s selection of the optimal thickness restored high satisfaction and function at 6-months. The median Forgotten Joint Score of 73 is expected to increase with prolonged follow-up and was between the 80 and 70 point cut-offs associated with negligible and minor knee function restrictions, respectively [21,29]. Similarly, the median Oxford Knee Score of 42 was in the excellent category range of 42–48 described by Kalairajah [22].

The present study has several limitations. First, the reliability of the insert goniometer’s orientation measurement could depend on the TKA conditions. The conditions in the present study included a mid-vastus exposure that promoted patella tracking, a medial ball-in-socket and lateral flat insert conformity like the native knee, PCL retention, and components set coincident to the patient’s pre-arthritic joint lines with KA. An insert with a less conforming medial compartment than a 1:1 ball-in-socket loses 2° of internal rotation at 90° flexion [19]. An insert with a more conforming lateral compartment than a flat articular surface does not rotate about a medial pivot point and rotates less [9,10,12]. PCL resection causes an 8° loss of internal rotation with flexion [20]. Accordingly, the insert goniometer could function differently with a parapatellar incision that detaches the vastus medialis, mechanical alignment (MA) and restricted KA that do not restore the patient’s pre-arthritic joint lines, PCL resection, and insert conformities that differ from the native knee. A second limitation is that there could have been differences in knee and PCL laxity between the 22 TKAs because of variability in the surgical skills and decision-making of the two surgeons. However, any differences did not affect the insert goniometer measurements as each TKA had an optimal insert thickness. Finally, one author is also the inventor of the insert goniometer and, therefore, might be prone to bias [30].

## 5. Conclusions

In summary, an insert goniometer is an inexpensive tool that, when used with a medial ball-in-socket implant design, provides quantitative guidance into personalizing ligament and PCL tension when performing unrestricted caliper-verified KA TKA. In addition, knowing the difference in tibial orientation in extension and at 90° flexion between insert thicknesses can identify the thickness with the maximum orientation at each knee position and help the surgeon select the optimal insert thickness within a tolerance of ±1 mm, which restores high function.

## Figures and Tables

**Table 1:** Preoperative Patient Demographics, Clinical and Radiographic Characteristics, and Pre-and Postoperative Function of Included Patients.

Preoperative Demographics and Clinical and Radiographic Characteristics	Included Patients *n* = 22	
	DEMOGRAPHICS	
Age (years)	72 (± 6)	
Sex (male)	11 (50%)	
Body Mass Index (kg/m^2^)	30 (±6.2)	
	PREOPERATIVE MOTION AND KELLGREN–LAWRENCE SCORE	
Extension (degrees)	10 (±7)	
Flexion (degrees)	112 (±8)	
Kellgren–Lawrence Score	3.6 (±0.6)	
	PREOPERATIVE FUNCTION	POSTOPERATIVE FUNCTION
Oxford Score (48 is best, 0 is worst)	Mean (±SD): 23 (±8)	Median (±SD): 42 (±6)
Knee Society Score	Mean (±SD): 34 (±17)	
Knee Function Score	Mean (±SD): 52 (±23)	
Forgotten Knee Score		Median (±SD): 73 (±29)
	RADIOGRAPHIC MEASUREMENTS	
	PREOPERATIVE	POSTOPERATIVE
Femoral-Tibial-Angle (+valgus/−varus)	11° (±3.1°)	2° (±3.2°)
DLFA (<90° valgus/>90° varus)		83° (±1.8°)
PMTA (<90° varus/>90° valgus)		85° (±2.1°)

DLFA: Distal Lateral Femoral Angle (using anatomic femur axis); PMTA: Proximal Medial Tibial Angle (using anatomic tibia axis).

## Data Availability

The data presented in this study are available on request from the corresponding author.
